# Mathematical modeling of thrombus formation in idealized models of aortic dissection: initial findings and potential applications

**DOI:** 10.1007/s00285-016-0986-4

**Published:** 2016-03-23

**Authors:** Claudia Menichini, Xiao Yun Xu

**Affiliations:** Department of Chemical Engineering, Imperial College London, South Kensington Campus, SW7 2AZ London, UK

**Keywords:** Aortic dissection, Computational fluid dynamics, Thrombosis, Shear stress, Residence time, 76Z05, 92C50, 76D05, 92-08

## Abstract

Aortic dissection is a major aortic catastrophe with a high morbidity and mortality risk caused by the formation of a tear in the aortic wall. The development of a second blood filled region defined as the “false lumen” causes highly disturbed flow patterns and creates local hemodynamic conditions likely to promote the formation of thrombus in the false lumen. Previous research has shown that patient prognosis is influenced by the level of thrombosis in the false lumen, with false lumen patency and partial thrombosis being associated with late complications and complete thrombosis of the false lumen having beneficial effects on patient outcomes. In this paper, a new hemodynamics-based model is proposed to predict the formation of thrombus in Type B dissection. Shear rates, fluid residence time, and platelet distribution are employed to evaluate the likelihood for thrombosis and to simulate the growth of thrombus and its effects on blood flow over time. The model is applied to different idealized aortic dissections to investigate the effect of geometric features on thrombus formation. Our results are in qualitative agreement with in-vivo observations, and show the potential applicability of such a modeling approach to predict the progression of aortic dissection in anatomically realistic geometries.

## Introduction

Aortic dissection is a critical medical condition caused by a tear in the inner surface of the aortic wall, the intima. The formation of a tear allows blood to flow in-between the inner and outer layers of the aortic wall in the so called “false lumen” (FL). This aortic catastrophe compromises the normal function of the circulatory system and is associated with high mortality rates. Possible complications include rupture, enlarging aneurysm, aortic insufficiency, or malperfusion syndromes (Erbel et al. 2001). Urgent surgical intervention is required if the entry tear is located in the ascending aorta (Stanford Type A dissection), as more complications can arise from this specific morphology. Medical treatments or endovascular repairs are typically adopted when the entry tear is situated in the descending aorta (Stanford Type B dissections), depending on the stability of the patient’s conditions.

The highly disturbed flow patterns and large variability in wall shear stress (WSS) observed in aortic dissection are likely to promote thrombus formation in the false lumen. For both endovascular and medical treatments, previous research has highlighted a connection between patient prognosis and the level of thrombosis in the false lumen, with partial thrombosis of the false lumen being associated with a higher mortality rate (Tsai et al. 2007). Complete thrombosis of the false lumen, on the other hand, has demonstrated to have beneficial effects on patients’ recovery owing to re-absorption of the dissected area (Bernard et al. 2001; Trimarchi et al. 2013). Therefore, it is important to understand which conditions would promote complete thrombosis of the false lumen, and mathematical modeling is a useful tool for this purpose.

Thrombus formation is a complex biological process which involves a large number of chemical and biological species, transport phenomena, and kinetic processes acting on different time scales. Several modeling approaches have been proposed in order to predict thrombosis and describe the different biochemical and physical mechanisms involved in the formation of blood clots. These approaches can be distinguished between hemodynamics-based and kinetics-based. Numerous experimental and computational studies have been conducted in order to qualitatively correlate thrombus formation and local flow features. The effects of local geometry and corresponding fluid dynamics on platelet activation and deposition were first investigated by Stein et al. (1977) and Karino and Goldsmith (1979), who showed a positive correlation between Reynolds numbers and thrombus formation. Schoephoerster et al. (1993) measured platelet density in idealized stenosis and aneurysms and observed maximum depositions in areas of flow recirculation and reattachment. Bluestein et al. (1997) developed a computational model to analyse the corresponding fluid dynamics in laminar and turbulent conditions and introduced a level of activation parameter to quantify the cumulative effect of shear stress variations and exposure time on platelet activation. The role of shear stress in platelet aggregation and activation was also investigated by Bark and Ku (2010) and Bark et al. (2012) who performed their experiments on stenotic vessels of varying degrees of stenosis. They showed positive correlation between thrombus accumulation rates and wall shear rates up to 6000 s$^{-1}$ and developed a computational model to predict thrombus growth and occlusion times in stenosed arteries (Bark et al. 2012; Bark and Ku 2013). The work of Biasetti et al. (2011) was focused on the relationship between coherent vortical structures and intra-luminal thrombus formation in fusiform and saccular aneurysm; then they used integrated transport equations to model the coagulation cascade and examine the distribution of chemical agonists (Biasetti et al. 2012). Other fluid dynamics-motivated approaches include computational studies focused on the effects of fluid residence time on thrombus formation in continuum and Lattice-Boltzman frameworks (Narracott et al. 2005; Harrison et al. 2007; Rayz et al. 2010), which highlighted a connection between low shear stress, high residence time, and thrombosis.

While hemodynamics-based studies are capable of demonstrating the relationship between fluid dynamics and thrombus formation, they neglect the complex network of biochemical mechanisms involved in the process and often fail to reproduce the growth of thrombus over time. More comprehensive models have been proposed by coupling fluid flow with chemical processes and including transport of different species. A large number of continuum models have been developed to simulate the complex mechanisms of the coagulation cascade, including the transport of biochemical components modeled as dilute species. The components are described by a set of convection-diffusion-reaction (CDR) processes and expressed through a system of ordinary or partial differential equations (ODEs or PDEs), coupled with Navier–Stokes and continuity equations to describe fluid flow (Sorensen et al. 1999; Anand et al. 2003; Goodman et al. 2005; Bedekar et al. 2005; Leiderman and Fogelson 2011; Biasetti et al. 2012). Comprehensive discrete and statistical models have also been proposed to simulate the transport of a finite number of particles within the system and their interactions (Fogelson and Guy 2008; Longest and Kleinstreuer 2003; Xu et al. 2008). However, the high computational costs of kinetics-based approaches confine the applicability of such models to either relatively simple geometries, or realistic geometries but without accounting for thrombus growth and its effects on the flow field (Bedekar et al. 2005; Biasetti et al. 2012).

The aim of this study is to develop an alternative modeling approach to predict thrombus formation in complex geometries and to give insight into the formation of thrombus in aortic dissection. In this paper, a new computational model is presented which is able to predict thrombosis in Type B aortic dissection and its effects on blood flow. Predictions are based on local hemodynamic parameters and the transport of a limited number of components. The model is applied to six phantom geometries of aortic dissection with different tear configuration and sizes to investigate the effect of geometric features on hemodynamics and thrombus formation in Type B aortic dissection. Our long-term objective is to provide a predictive tool for patient-specific studies.

## Methodology

The evolution of aortic dissection causes significant morphological changes in the aorta. The formation of intimal tears and a false lumen tends to lead to highly disturbed flow, including turbulence, recirculation zones, and large variation in wall shear stress. All these parameters have a major influence on thrombus formation within the false lumen. The tearing of the aortic wall, the high shear stress levels detected in the proximity of tears, and the existence of shear micro-gradients can potentially promote the initial activation of platelets and the formation of platelet aggregates (Hellums 1994; Nesbitt et al. 2009; Sheriff et al. 2010). At the same time, the formation of recirculation zones within the false lumen characterized by stasis, long residence times, low shear stress and therefore high viscosity together with the potential thrombogenicity of the subendothelial tissue create an ideal environment for platelet aggregation and deposition, leading to thrombus formation on the walls (Woolf 1998; Malek et al. 1999; Goel and Diamond 2002). Based on these considerations, a new computational model is developed with the aim to predict thrombus formation in aortic dissection. Blood flow is simulated by means of computational fluid dynamics (CFD) and is integrated with mass transfer equations to model thrombus growth. Areas with high probability for thrombus growth are identified through the evaluation of local shear rates, residence time, and distribution of activated platelets. The number of species modeled is significantly reduced as compared with models of the coagulation cascade, and time scales of thrombus growth have been adapted to facilitate simulation of the system in a short time frame.

### Geometry

**Fig. 1:**
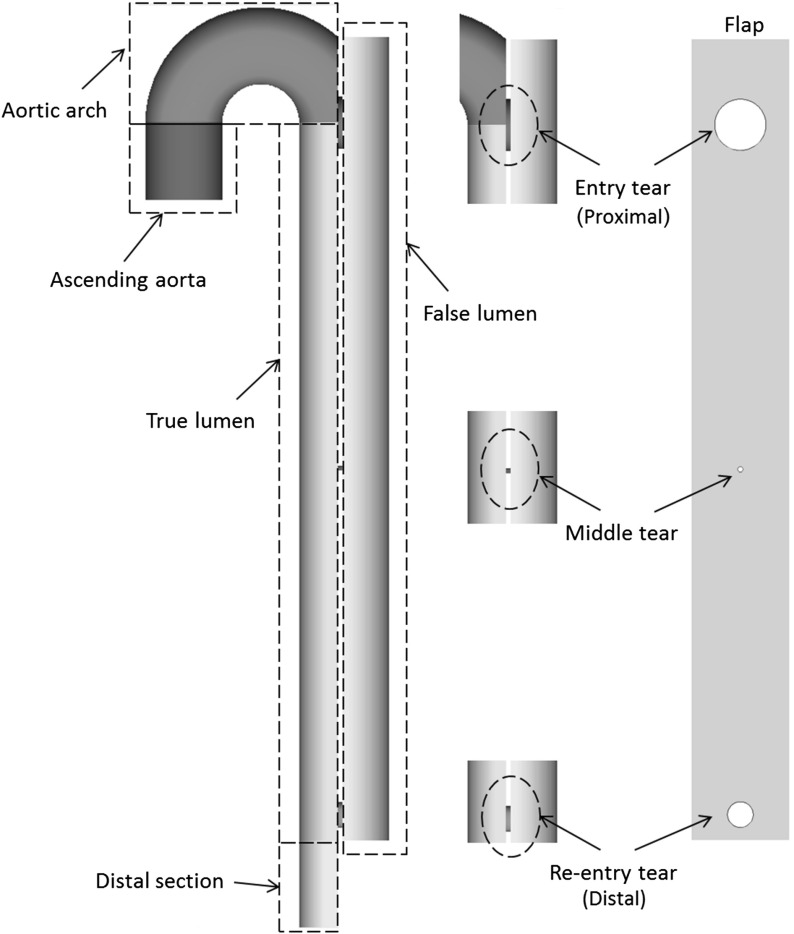
Hypothetical phantom for aortic dissection used in this study. Tear size and configurations are varied in the different models analysed

Six different 2D phantom models of aortic dissection have been generated in SolidWorks for this study. The geometric features of the models are presented in Fig. 1 and Table 1. Tear size and location have been varied in order to analyse different scenarios and investigate the influence of morphological features on the formation of thrombus in aortic dissection. The six phantom models were discretized into a block-structured grid made of hexahedral elements using ICEM (Ansys Inc.). For each of the 2D geometries created, a mesh sensitivity test has been carried out, and a mesh consisting of 53,000–56,000 elements depending on the number and size of the tears was found to be adequate for mesh independent solutions.

One additional geometry was created for a benchmark test reproducing the experimental set-up proposed by Taylor et al. (2014). It consists of a 3D backward facing step of circular cross section, with a diameter of 10 mm and a step height of 2.5 mm. A fine structured mesh was generated, consisting of approximately 1.2 million hexahedral elements.

**Table 1:** Geometric features of the phantom models analysed in this study, with maximum peak shear stress and time-averaged wall shear stress (TAWSS), and percentage of flow diverted into the false lumen. The flow into the false lumen is calculated at peak systole

	No. of tears	Diameter (mm)		Max WSS (Pa)	Max TAWSS (Pa)		Flow % into FL
		PT	DT		PT	DT	
C1	1	10	n/a	55	9	n/a	$\sim 0$
C2	1	n/a	10	37	n/a	5.8	$\sim 0$
C3	2	10	5$^{\mathrm{a}}$	120	18	24	26
C4	2	10	5	138	20	24	35
C5	2	10	10	128	12.5	18	37
C6	2	20	10	120	15	19	41

*PT* proximal tear, *DT* distal tear, *FL* false lumen

$^{\mathrm{a}}$ Re-entry tear placed in the middle section

### Model development

The model predictions are based on the following assumptions: (1) cell adhesion can only occur in regions where wall shear rates fall below the physiological threshold of 100 s$^{-1}$ (Goel and Diamond 2002); (2) the concentration of coagulation factors is high in regions of flow recirculation and stasis, characterized by long residence times, stabilizing the forming thrombi (Nesbitt et al. 2009). These assumptions allow us to focus on the transport of a limited number of species and to evaluate the growth of thrombus based on average parameters, significantly reducing the overall computational cost of the model. We assume that the initial activation of platelets is triggered by the inflammatory response caused by the splitting of the aortic wall and by the high shear stress experienced in the tear regions (Nesbitt et al. 2009; Sheriff et al. 2010), where the local time-averaged shear stress (TAWSS) can reach values above 40 Pa (Cheng et al. 2015). The initial platelet activation is accounted for through a 5 % background activation level (see Sect. 2.2—*Platelet Activation*). The main features of the model and key species transported are described below.

*Residence time (RT)* This variable is used to identify regions of stagnancy and flow recirculation, which are associated with an increased likelihood for thrombus formation. RT is modeled as a tracer passively transported with the flow and obeying to the following equation (Ghirelli and Leckner 2004):

1 $$\begin{aligned} \frac{\partial RT}{\partial t} + \mathbf{\textit{v}} \cdot \nabla RT = D_{RT} \nabla ^{2} RT + 1 \end{aligned}$$

where *t* is time, *v* is the velocity of blood, $D_{RT}$ represents the self-diffusivity of blood ($D_{RT}$ = 1.14 $\times 10^{-11}$ m$^{2} \mathrm{s}^{-1}$, Harrison et al. 2007) and the source term considers a unit increase in RT for each unit increase in time. As the absolute residence time is a function of time, the increase in RT for each cardiac cycle has been normalized with respect to cycle period and its non-dimensional counterpart is called relative residence time (RRT).

*Resting and activated platelets (RP and AP)* are modeled as dilute chemical species passively transported with the flow, which can be described by a convection-diffusion-reaction transport equation (Sorensen et al. 1999):

2 $$\begin{aligned} \frac{\partial c_i}{\partial t} + \mathbf{\textit{v}} \cdot \nabla c_i = D_p \nabla ^{2} c_i + S_i ~~~~~ i = AP, RP \end{aligned}$$

where $c_i$ represents the concentration of platelets (RP or AP). The diffusivity $D_p$ accounts for the shear-enhancing effect of RBCs through the diffusivity model proposed by Wootton et al. (2001):

3 $$\begin{aligned} D_p = D_{p_t} + \alpha \dot{\gamma }\end{aligned}$$

where $D_{p_t}$ is the thermal diffusivity ($D_{p_t}$ = $1.6\times 10^{-9}$ $\mathrm{cm}^{2}/\mathrm{s}$) and the coefficient $\alpha$ is equal to $7 \times 10^{-9} \mathrm{cm}^{2}$ (Wootton et al. 2001). The reactive source term $S_i$ is the sum of two different contributions that model the effect of APs on the further activation of RPs ($r_1$) and activation by thrombin exposure ($r_2$) (Anand et al. 2003). As the transport of thrombin is not included in the model, it is assumed that the concentration of thrombin would be higher in areas of high residence time. Therefore, the value of $r_2$ has been scaled by RRT:

4 $$\begin{aligned} r_{1} = k_{1} [AP] [RP] \qquad \qquad r_{2} = k_{2} [RP]~ RRT \end{aligned}$$

$k_{1}$ and $k_{2}$ are the kinetic constants, whose values presented in Table 2 have been taken from Anand et al. (2003). To avoid numerical ill-conditioning, the concentrations [AP] and [RP] have been normalized against their initial values.

The local concentration of AP and RP should be interpreted as a measure for platelet activity rather than a specific value (Papadopoulos et al. 2014). High AP levels represent a higher probability to encounter activated platelet in a specific region, which is a key element to allow the formation of thrombus.

*Platelet activation* The longitudinal tearing of the aortic wall layers is likely to cause an inflammatory response, which may stimulate platelet activation. Additionally, as particularly high shear rates are generally observed in proximity to the tears, we hypothesise that a shear-induced process might also be involved in the activation of platelets leading to false lumen thrombosis. For these reasons, a background activation level equal to 5 % of the inlet concentration of resting platelets (Sorensen et al. 1999) is assumed as an initial condition, which allows us to neglect the initial activation process and lag times, and focus on the propagation of platelet activation, described by the reaction rates $r_1$ and $r_2$.

Multiple authors have observed a threshold response of platelet activation to shear stress, dependent on both exposure time and shear magnitude (Hellums 1994; Sheriff et al. 2010). As the tear regions are characterised by low exposure times due to a fast flowing stream of blood, it is reasonable to assume that, for aortic dissection, the peak shear observed in this area will have the strongest impact on shear-induced platelet activation. A shear rate threshold of 10,000 $\mathrm{s}^{-1}$ (equivalent to a shear stress of $\sim 40$ Pa based on the Quemada model) is used in this study to evaluate the likelihood for shear-induced platelet activation at low exposure times (Holme et al. 1997).

*Coagulant (C) and bound platelets (BP)* The variable coagulant (C) accounts for the processes involved in the coagulation cascade, and is formed on the walls where the local time-averaged shear rate is lower than a fixed threshold. The formation of coagulant on the walls in the model is associated with tissue factor exposure and interaction of cells with fibrinogen, which is enhanced at low shear rates (Savage et al. 1996). Its diffusion in the bulk is related to the propagation of coagulation factors and thrombin generation. As several studies report the occurrence of thrombosis for shear rates below 100 s$^{-1}$ (Goel and Diamond 2002; Malek et al. 1999), a local time-averaged wall shear rate threshold of 90 s$^{-1}$ has been chosen for this study. Although the minimum shear rate might have a stronger impact on thrombus formation, this value is more sensitive to the rheological model used and is affected by the upstream flow, which is subject to variation during the long time scale of thrombus growth of weeks or months. For this reason, time-averaged values are considered more reliable and robust, which are used to determine coagulant formation on the wall. In the model presented the coagulant promotes the coagulation of activated platelets and the formation of thrombus, defined through a static variable called bound platelets (BP).

The formation of blood clots is defined by a complex chain of biochemical reactions taking place on the surface of a growing thrombus. While the transport of proteins and coagulation factors in the bulk is convection-dominated, in the near-wall areas and in zones of low velocity and high residence time the transport of clotting factors is diffusion-limited. The growth rate is therefore determined by the slow diffusion of coagulation factors to the thrombus surface, which controls the surface reaction rates. The large difference in time-scales between processes taking place on the macro-scale and micro-scale makes it extremely challenging to simulate the growth process over time. To circumvent this problem, the different time-scales of convection-dominated flow and diffusion-limited surface reactions are considered separately. This is achieved by modeling the transport of coagulant via a shear-dependent diffusive mechanism. This represents a moving boundary condition emulating surface reactions taking place on the advancing front of a growing thrombus. The effects of convection on surface reactions are considered through a coefficient $\phi _{\dot{\gamma }}$ (defined later). The transport equations for C and BP are the following:

5 $$\begin{aligned} \frac{\partial C}{\partial t}= & {} D_{c_{eff}} \nabla ^{2} C + k_c ~\phi _{C}~ \phi _{\dot{\gamma }} \end{aligned}$$

6 $$\begin{aligned} D_{c_{eff}}= & {} \phi _{\dot{\gamma }} D_c \end{aligned}$$

7 $$\begin{aligned} \phi _{C}= & {} \phi _{C}(BP) \end{aligned}$$

8 $$\begin{aligned} \frac{\partial BP}{\partial t}= & {} k_{BP} ~\phi _{BP} ~ \phi _{\dot{\gamma }} ~ [AP] \end{aligned}$$

9 $$\begin{aligned} \phi _{BP}= & {} \phi _{BP}(C,RRT) \end{aligned}$$

The coagulant is initially formed on the walls in low shear regions. When the local values of C, AP, and RRT are sufficiently high, BP will be formed and will activate a feedback mechanism promoting further formation of coagulation factors. $\phi _{C}(BP)$ and $\phi _{BP}(C,RRT)$ are coefficients which can assume values between 0 and 1, defined by simple saturation (or switching) functions (Eq. 10). These functions can gradually turn on and off the thrombus formation process depending on the local environment.

10 $$\begin{aligned} \phi = \prod \frac{X_i^2}{X_{i}^2+X_{i_t}^2} \end{aligned}$$

If the local concentration of any of the variables involved in the process $$X_i$$ is at least 2–3 times smaller than a defined threshold $$X_{i_t}$$ ($$ RRT_{t} = 0.9, C_{t} = 10$$ nmol and $$BP_{t} = 20$$ nmol), the reaction will stop. The saturation functions prevent the occurrence of sharp changes in concentration and avoid numerical instabilities related to the use of discontinuous step functions. The coefficient $$\phi _{\dot{\gamma }}$$ was introduced in the model to control transport and formation of coagulant and prevent the formation of thrombus in high shear regions, which would be physically unrealistic. $$\phi _{\dot{\gamma }}$$ is a function of the local time-averaged shear rate $$\bar{\dot{\gamma }}$$ and is defined as:

11 $$\begin{aligned} \phi _{\dot{\gamma }} = \frac{\bar{\dot{\gamma }}_t^2}{\bar{\dot{\gamma }}^2+\bar{\dot{\gamma }}_t^2} \end{aligned}$$

where the bulk shear rate threshold $\bar{\dot{\gamma }_t}$ in Eq. 11 is fixed at 100 s$^{-1}$ in this study. The coefficient $\phi _{\dot{\gamma }}$ assumes a maximum value of 1 corresponding to no flow conditions and tends to a minimum of 0 for very large shear rates. For low values of $\phi _{\dot{\gamma }}$, the convective flow will oppose the deposition of blood cells on the thrombus surface and cause washout of coagulation factors. Conversely, for low bulk shear rates the adhesion strength between platelets will be stronger than the tangential force exerted by the flow. This measure was introduced in the model in order to account for the effects of blood flow on growth while overcoming the issues related to the difference in time scales between diffusion-limited biochemical reactions and convection-dominated blood flow, allowing reasonable simulation times.

*Momentum source* The momentum balance is described by the Navier–Stokes equations. Following the approach proposed by Leiderman and Fogelson (2011), the effects of a growing thrombus on blood flow are modeled by introducing in the momentum equations a negative source term proportional to the concentration of BP. This source term represents a fictitious force opposing fluid motion in regions where thrombus is growing. The adapted equation is reported below:

12 $$\begin{aligned} \rho \frac{\partial \mathbf{u}}{\partial t} + \rho \mathbf{u} \cdot \nabla \mathbf{u}= & {} - \nabla p + \nabla \cdot (\mu (\nabla \mathbf{u}+(\nabla \mathbf{u})^{T})) - S_{M} \end{aligned}$$

13 $$\begin{aligned} S_M= & {} k_M \frac{BP^{2}}{BP^{2} + BP_{t}^{2}} \mathbf{u} \end{aligned}$$

Once again, a saturation function has been introduced in the source term rather than a step function to avoid numerical instabilities. The value chosen for $k_M$ is large enough to stop the flow where thrombus is formed (see Table 2).

*Viscosity variation* Within in a growing thrombus, blood viscosity is allowed to change to simulate increased resistance. A viscosity function was introduced for this purpose accounting for an increase up to 100 fold normal viscosity values (Goodman et al. 2005), proportionally to the concentration of BP (Eq. 14). The viscosity of blood $\mu$ only deviates from its reference value $\mu _{0}$ in regions with a BP concentration greater than 0.

14 $$\begin{aligned} \mu = \mu _{0} \left( 1+ 100 \frac{BP^{2}}{BP^{2} + BP_{t}^{2}}\right) \end{aligned}$$

**Table 2:** Rate constants used in the computational model

	Value	Units	References
$k_1$	0.5	s$^{-1}$	Anand et al. (2003)
$k_2$	$1.2 \times 10^{-8}$	$\mathrm{mL/platelets}\cdot \mathrm{s}$	Anand et al. (2003)
$k_c$	200	$\mathrm{nmol/L}\cdot \mathrm{s}$	
$k_{BP}$	$10^{a}$	$\mathrm{nmol/L}\cdot \mathrm{s}$	
$k_M$	$10^{7}$	$\mathrm{kg/m}^{3}\mathrm{s}$	

$^{\mathrm{a}}\,k_{BP}$ refers to a concentration [AP] normalized against its initial value $1.25\times 10^{7}$ platelets/mL

### Flow model, initialisation, and boundary conditions

The model has been implemented in Ansys CFX 15 (Ansys Inc.) via user defined subroutines and initialized with a physiological concentration of resting platelets (2.5 $\times \, 10^{8}$ platelets/mL, Wootton et al. 2001), zero for RT, BP, and C, and a background activation level equal to 5 % of the inlet concentration of RP has been prescribed (Sorensen et al. 1999). The flow was initialized by simulating the system for two cardiac cycles in order to achieve a periodic solution, and the thrombus model was introduced only from the third cardiac cycle. Blood is modeled as a non-Newtonian fluid described by the viscosity model proposed by Quemada (1978). As Reynolds numbers in large vessels are generally below the threshold for turbulent flow, blood flow is assumed to be laminar (Fung 1997).

A realistic aortic flow waveform was applied at the model inlet, together with a flat velocity profile (Cheng et al. 2014, Fig.2). The waveform presents a frequency of 1.02 Hz, maximum velocity 0.51 m/s and a Womersley number of 18.8. A no-slip condition was imposed at the rigid walls, with (1) specified concentration of C at 100 or 0 nmol/L depending on the local shear rate; (2) flux boundary condition described through a first order adhesion function for RP and AP (Wootton et al. 2001) in areas of low shear ($\bar{\dot{\gamma }} ~ <~ 90 ~ \mathrm{s}^{-1}$); and (3) zero flux for other species. Only on the aortic arch a zero flux boundary condition was adopted for all variables to avoid thrombus formation in artificial recirculation zones created by the exclusion of the aortic branches.

**Fig. 2:**
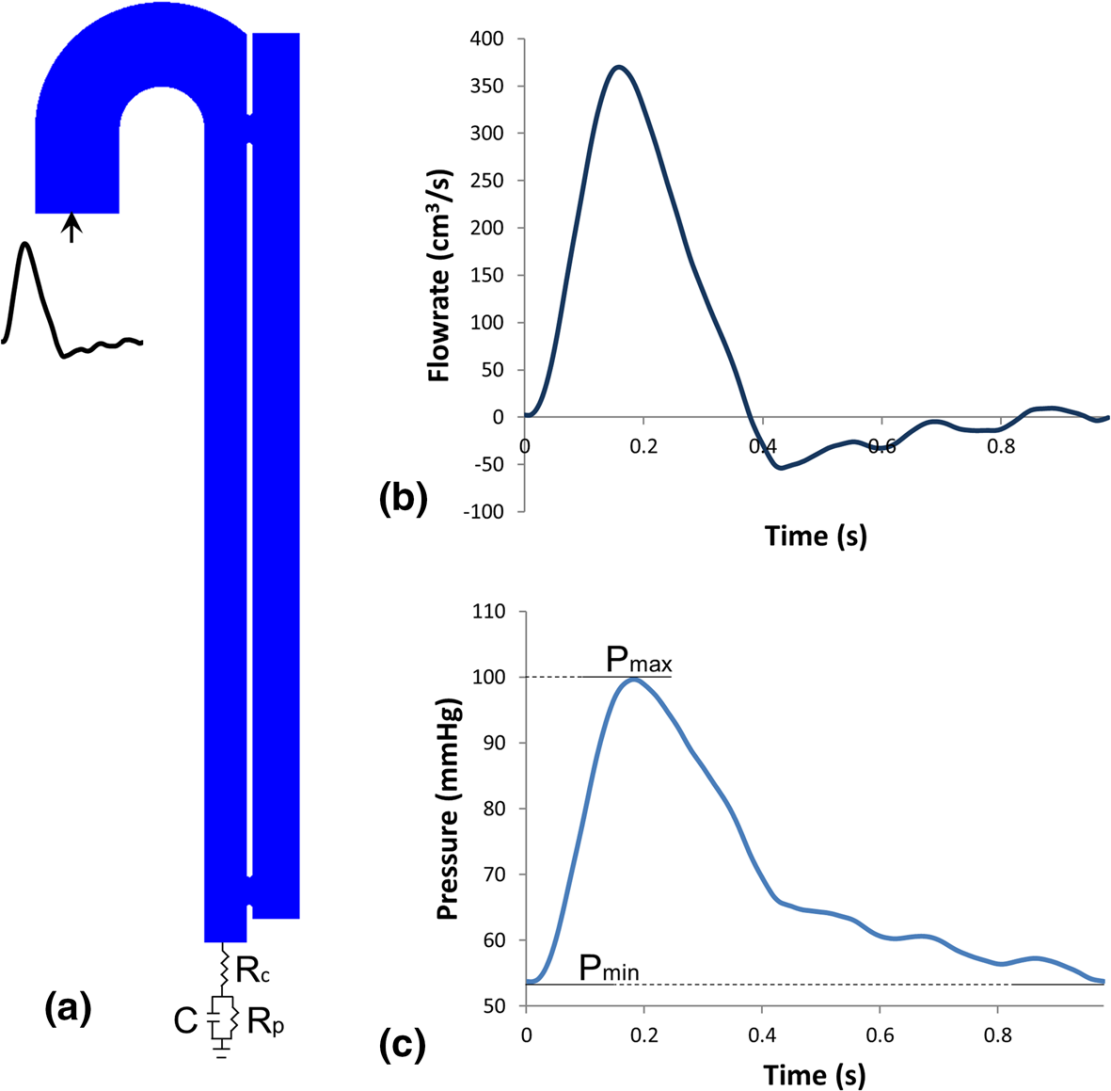
**a** Geometric model with *inlet* and *outlet* boundary conditions. $R_c$ impedence of the aorta, *C* compliance of aortic walls, $R_p$ resistance of peripheral vessels. **b** Flow waveform applied at the *inlet*. **c** Pressure waveform calculated from the 3-element Windkessel model, implemented as *outlet* boundary condition. A lag time of 0.024 s exists between pressure and flow waveform

A pressure waveform calculated through a three-element Windkessel model was applied at the outlet (Fig. 2c). For the three-element model, the system can be represented by the electrical circuit presented in Fig. 2, where the potential represents the arterial pressure, the current the flowrate, and impedence of the aorta, wall compliance, and peripheral resistance of the vessel are respectively represented by $R_c$, C, and $R_p$ (Ruel and Lachance 2010). By solving the circuit using the analogies between pressure, flowrate, potential, and current, the following equation is obtained:

15 $$\begin{aligned} P = Q ~ (R_p + R_c) +C R_p R_c \frac{dQ}{dt} - C R_p \frac{dP}{dt} \end{aligned}$$

The fitting parameters C, $R_p$, and $R_c$ have been calculated by matching the velocity waveform with the pressure measurements reported in the literature for the distal region in aortic dissection (Alimohammadi et al. 2014), and minimizing the difference between experimental measurements and computational solution through a user-developed Matlab code.

A sensitivity analysis has been carried out in order to calibrate the kinetic constants of the model. The sensitivity test has shown that as thrombus formation is originated in areas of high residence time, the extent of thrombus is independent of the kinetic constants $k_c$ and $k_{BP}$, which regulate how fast the threshold concentrations $C_t$ and $BP_{t}$ are reached. In fact, as thrombus growth occurs in regions of slow flow, clot formation only causes gradual changes to the flow field. This has allowed us to artificially accelerate the process so that evidence of thrombosis could be observed in a reasonably short time frame (20 cardiac cycles). A time-step sensitivity test has also been performed, and a value of 0.005 s was found to be sufficient for an acceptable numerical accuracy. Each simulation required approximately 24 h on a 16.0 GB RAM desktop PC with Intel Core i7-2600 3.40 GHz. The results have been analysed through the post-processing software CEI Ensight 10 (CEI Inc., Apex, NC, USA).

## Results

### Benchmark test: thrombus formation in backward facing step

To test the performance of the model before applying it to the dissection geometries, thrombus formation in a backward facing step was simulated, so as to reproduce the conditions adopted by Taylor et al. (2014) and compare with their experimental results. A fixed flowrate of 0.76 L/min was applied at the inlet, and a zero pressure was assumed at the outlet. The Quemada viscosity model was adapted to incorporate the reported hematocrit of 30 % (Marcinkowska-Gapiska et al. 2007). As the flow is non-pulsatile, a lower wall shear rate threshold for thrombus formation of 1 s$^{-1}$ was adopted, which is consistent with values reported by Polanczyk et al. (2015) for blood hematocrit near 30 %. The bulk shear rate threshold $\bar{\dot{\gamma }_t}$ was set to 10 s$^{-1}$, while all the other parameters and boundary conditions were left unchanged. The simulation required approximately 86 h for a time step of 0.01 s and a total simulation time of 50 s.

**Fig. 3:**
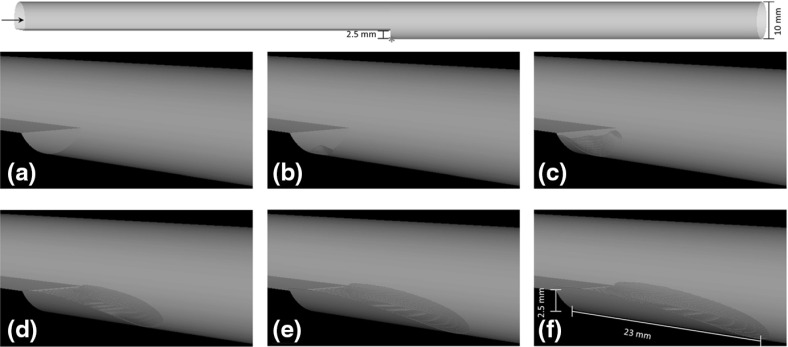
Predicted thrombus formation (*red*) in the BFS geometry at **a** t $=$ 1 s, **b** t $=$ 7 s, **c** t $=$ 14 s, **d** t $=$ 28 s, **e** t $=$ 39 s, **f** t $=$ 50 s. The *asterisk* shows the location where thrombus growth is started

Figure 3 shows the predicted thrombus formation as a function of time. Initial growth takes place downstream of the step in both axial and radial directions. Radial growth is stopped when the thrombus height reaches the step height (2.5 mm) and a maximum width equal to that of the step. After the thrombus reaches a total length of approximately 8 times the step height, growth is significantly slowed down, reaching a maximum length of about 23 mm. These observations are in good agreement with the experimental results of Taylor et al. (2014).

### Wall shear distribution and platelet activation

The complex geometry of aortic dissection allows a wide spectrum of shear rates on the walls. Particularly high shear stress is observed in the tear region, where a high-velocity jet of blood enters the false lumen. The time-averaged and maximum shear stress values are reported in Table 1. As it can be observed, these values are affected by the respective sizes and location of tears. Smaller tears and larger distances between proximal (near the aortic arch) and distal (placed in the distal section) tears are related to higher peak and time-averaged shear values. This result implies a higher likelihood for platelet activation in the initial stages of dissection, where the size of the tears is generally small, and in dissections presenting a large distance between tears. In most of the cases studied the peak shear stress for both tears is above the defined activation threshold of 40 Pa. The only exception is model C2 which has no primary tear and was designed to simulate a stented aortic dissection.

**Fig. 4:**
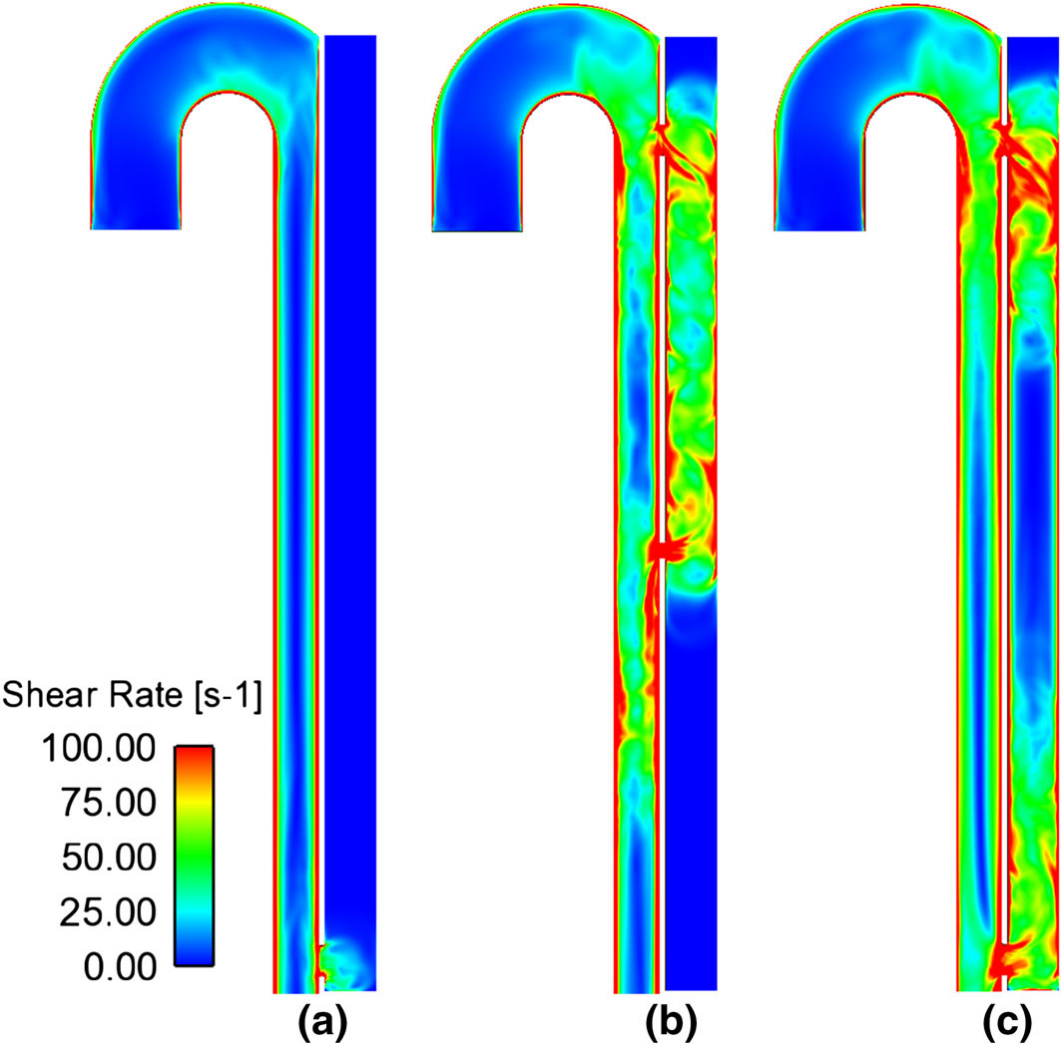
Cycle-averaged shear rates in models **a** C2, **b** C3, **c** C5

The size and location of tears also affect the distribution of low shear areas, and regions of stagnancy and flow recirculation are characterized by particularly low shear rates on the walls and high RT, as shown in Figs. 4 and 6. The local shear stress in these areas can possibly fall below the yield stress of blood, which represents the minimum shear necessary to prevent cell aggregation and cell-fibrinogen interaction, allowing blood to start flowing (Replogle et al. 1967). For larger openings and multiple connecting tears, the extent of these regions is reduced, with a lower probability for flow recirculation, stasis, and a long residence time, which would promote a buildup of coagulation factors and possibly lead to thrombosis.

### Distribution of residence time and activated platelets

**Fig. 5:**
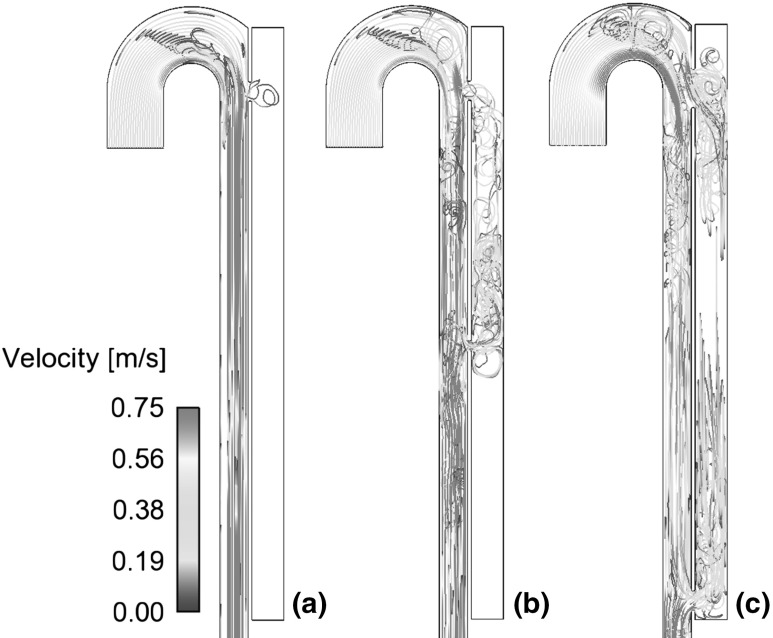
Pathlines of particle emitted in mid-systolic acceleration phase from the inlet section and tracked for 15 cardiac cycles in **a** C1, **b** C3, **c** C6

**Fig. 6:**
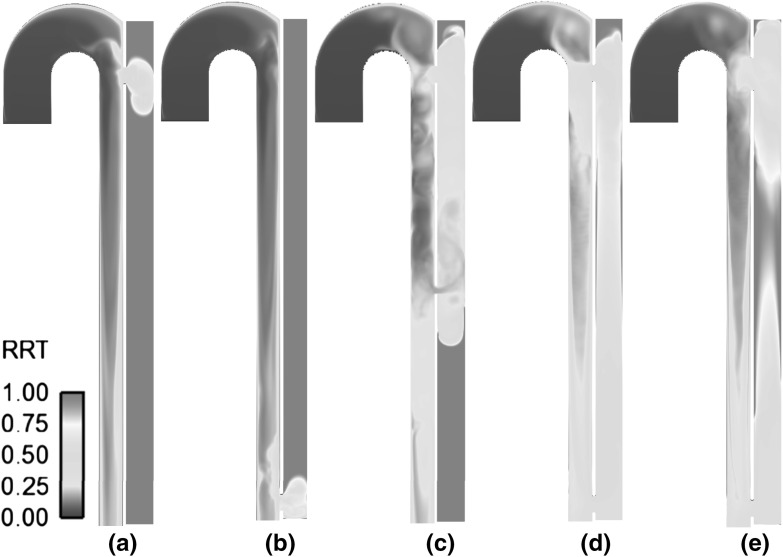
Cycle-averaged residence time distribution in different geometric models, normalized over time. **a** C1; **b** C2; **c** C3; **d** C5; **e** C6

The size, number, and location of the entry and re-entry tears affect the amount of blood entering the false lumen, local velocities, and flow patterns. For smaller proximal and distal connections, a reduced amount of blood is diverted into the false lumen (Table 1) resulting in lower local velocities and higher residence times. This pattern can be clearly observed by comparing the particle pathlines and time-averaged residence time distributions in the different geometries presented in Figs. 5 and 6. The models containing both a proximal and a distal tear exhibit flow recirculation and high residence time in the top and bottom regions of the false lumen and in-between the two tears (Figs. 5b, c, 6c–e). During a cardiac cycle, the two tears alternate their roles as entry and re-entry tears causing an inversion of the flow in the middle of the false lumen, reflected in a high residence time in the models with a large distance between tears (Fig. 6d, e). However, flow in the mid-section of the false lumen is organised, as shown by the linear trajectories in Fig. 5c, allowing normal physiological wall shear rates (Fig. 4) which prevent the occurrence of thrombosis in the idealized models. For smaller connections and reduced tear distance, larger recirculation zones with a high RT are observed in the top and bottom regions of the false lumen (Fig. 6c, d). The models containing only one proximal or distal tear present a long residence time in the entire false lumen, with slow flow recirculation and stagnant flow (Figs. 5a, 6a, b).

**Fig. 7:**
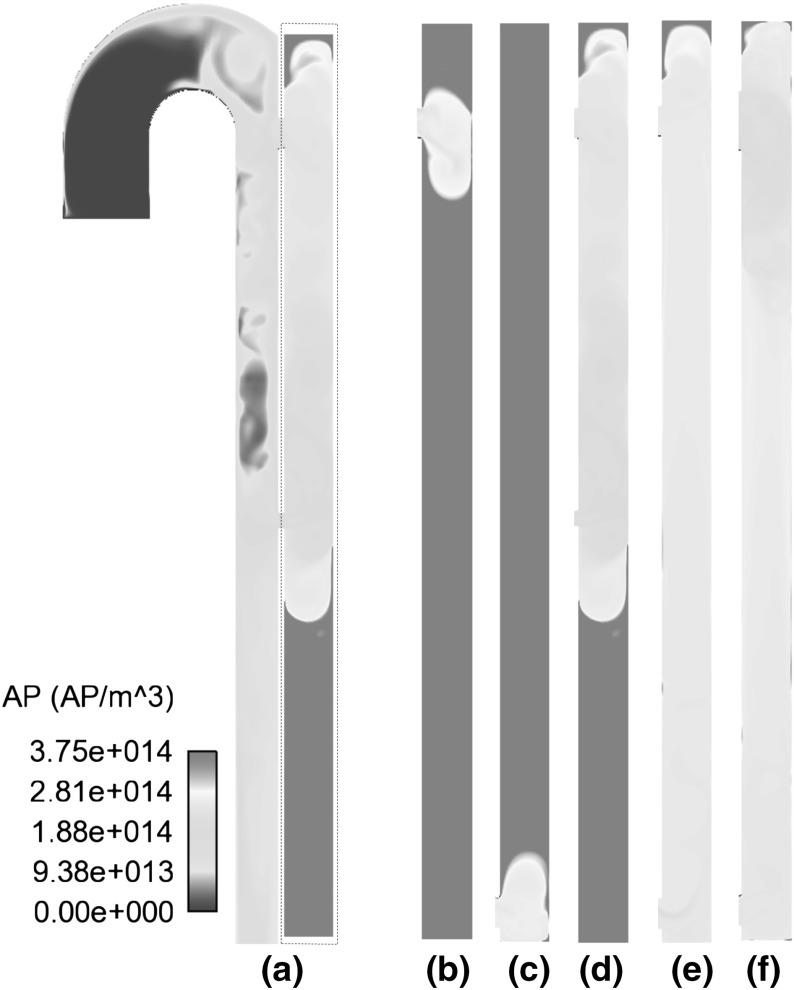
Distribution of AP in C3 (**a**), averaged over a full cardiac cycle, and false lumen details of: **b** C1; **c** C2; **d** C3; **e** C5; **f** C6

Similar observations can be made regarding the distribution of activated platelets. For all models, the highest concentration of AP is also located in the false lumen, implying higher platelet activity in this region. Local peaks are observed in areas of higher residence time and flow recirculation (Fig. 7). This higher concentration can be attributed to the partially stagnant flow and high residence time in the FL, which allows longer reaction time. The discharge of blood flow from the FL into the true lumen (TL) also causes higher AP concentrations around the tear regions in the TL (Fig. 7a).

### Thrombus growth over time

**Fig. 8:**
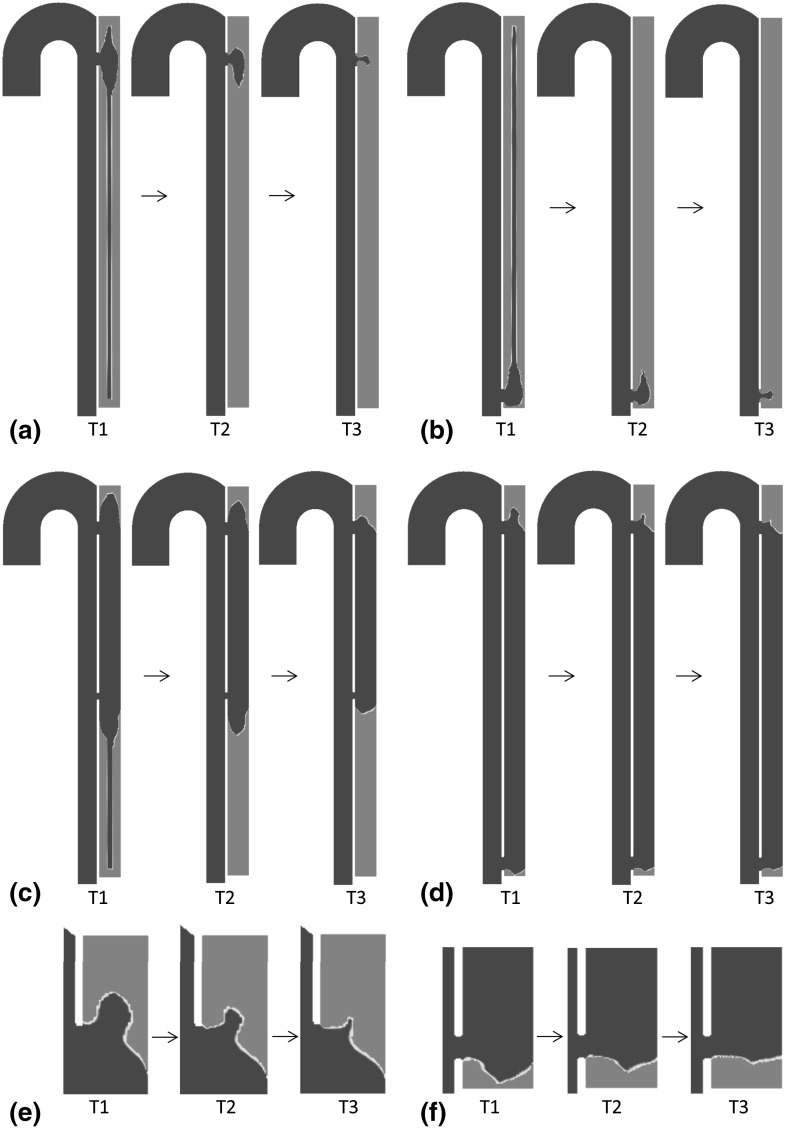
Thrombus growth over time in the six different geometric models. In *red*, areas where the concentration of BP is above the threshold fixed to activate the momentum sink. In *blue*, areas where the concentration of BP is zero. T1 $=$ 10 s; T2 $=$ 15 s; T3 $=$ 20 s. **a** C1; **b** C2; **c**; C3; **d** C5; **e** detail in *top* FL region in C6; **f** detail in *bottom* FL region in C4

The threshold for platelets adhesion is set at 90 s$^{-1}$. As expected, for all the geometric models thrombus growth is observed in areas of recirculating flow and stagnancy, where flow patterns follow chaotic trajectories and wall shear stress is low. The thrombosed region rapidly expands over time and is shaped by the flow features. For the models presenting both a proximal and distal tear, the thrombus is confined to top and bottom areas of the FL, while in the one-tear models the entire FL is exposed to clotting. When the thrombus reaches the tears, growth slows down significantly and eventually stops due to the high shear rates in the bulk caused by the high-velocity jet of blood which crosses the tears (Fig. 8). All these observations are in qualitative agreement with in vivo studies performed on patient-specific follow-ups (Cheng et al. 2013).

## Discussion

The progression of false lumen (FL) thrombosis has been recognised to have a major influence on patients’ survival and therapeutic outcomes. Bernard et al. (2001) and Tsai et al. (2007) identified FL patency as a key parameter to predict the progression of aortic dissection and underlined a link between evolution of the aortic diameter and FL patency and thrombosis. A positive connection was found by the authors between partial thrombosis and aortic expansion, justified by the possible increase in FL pressure caused by the formation of thrombi, which could lead to occlusion of the distal tear and further increase the risk for enlargement and rupture. On the other hand, complete FL thrombosis is generally considered as a positive sign for improved outcomes (Tsai et al. 2007; Trimarchi et al. 2013), and the effectiveness of endovascular and medical treatments is associated with spontaneous FL thrombosis.

Several models have been proposed in the literature for prediction of thrombus formation, which incorporate one or more stages of the coagulation cascade (Sorensen et al. 1999; Leiderman and Fogelson 2011; Anand et al. 2003; Goodman et al. 2005). The application of such models has been limited to simplified geometries and has required a number of assumptions to reduce computational costs, such as constant velocity and Newtonian behaviour of blood. Moreover, the effects of thrombus growth on fluid flow are often neglected. These assumptions limit the applicability of existing models to clinically relevant studies where the pulsatility of blood flow, the complexity of the geometry, and the effects of thrombus growth on the fluid domain need to be considered to obtain physiologically relevant predictions. This is further complicated by the difference in time scales between biochemical reactions, thrombus growth, and cardiac cycles which requires small time steps and large computational meshes to obtain robust solutions, increasing the overall computational cost.

The main aim of this study is to develop a feasible computational model that can be used to investigate the relationship between thrombus formation and hemodynamics in complex geometries and to predict the possible evolution of false lumen thrombosis in patients affected by Type B aortic dissection. As simulating the the process of thrombosis in aortic dissection over realistic time scales of days/months would be computationally prohibitive, the process has been artificially accelerated to allow simulation over a short time frame of 20 cardiac cycles. This acceleration is possible as FL thrombosis usually occurs in regions of low velocities, so that the formation and growth of thrombus only cause gradual changes in the flow field. Areas of high probability of thrombus formation are identified through cycle-averaged parameters, allowing us to simulate the overall growth process in a short time frame, thereby overcoming the limitations related to the large difference in time scales between biochemical reactions and transport processes. We consider this approach preferable to kinetics-based models which usually assume steady flow, as the pulsatile nature of blood flow and the large difference between systolic and diastolic velocities in the aorta affect the distribution of hemodynamic parameters. The use of cycle-averaged rather than steady parameters provides therefore a more accurate representation of the flow field and allows us to identify regions of low shear with more confidence. Our preliminary sensitivity study has indicated that the low shear rate threshold is the most sensitive parameter to define the regions of growth. A local wall shear rate threshold value of 90 s$^{-1}$ is chosen to initiate platelet adhesion in the phantom models under pulsatile flow, while a threshold of 1 s$^{-1}$ is used in the BFS geometry for a fixed inlet velocity. Further validations will be needed to determine the most appropriate value for this threshold in physiologically realistic pulsatile and static flow conditions. A comprehensive sensitivity analysis will also be required to determine the importance of the other model parameters.

Size and number of tears and distance between the tears affect local flow patterns and the development of areas of recirculation and stasis. The effects of geometric features on the distribution of activated platelets, residence time, and thrombus formation have been investigated and our results show that smaller tears increase the likelihood for thrombosis, as the percentage of flow diverted into the FL is lower resulting in more extended recirculation zones. Smaller tears are also associated with higher local shear stress, which is more likely to promote platelet activation. In the models containing only one proximal or distal tear the entire FL is exposed to very high residence time. However, in these models the local shear stress on the tears could be lower than the platelet activation threshold, such as model C2, presenting only one distal tear to simulate post-stenting geometry. In this case, the initial tearing of the aortic wall and the high shear rates experienced by cells pre-stenting are still considered likely to stimulate platelet activity, justifying the background activation level applied and the subsequent thrombus growth.

The computational model proposed in this study allows the simulation of thrombus growth over time. The extent of the thrombosed area is determined by the flow features and varies in different geometric models depending on the size and configuration of the tears. Previous studies have shown a possible correlation between low shear, high relative residence time, flow patterns, and thrombosis (Cheng et al. 2013), which is well captured in the present model. Our preliminary results show good qualitative agreement with previous in vivo studies and are consistent with the patient-specific case study analysed by Cheng et al. (2013) where an aortic dissection patient was monitored over a period of three years. In all our models, thrombus begins to form in the top of the FL where wall shear stress is particularly low due to flow recirculation, and gradually expands to the entire region above the entry tear. The possibility of thrombosis in other regions is determined by the tear configuration, which affects the flow patterns in FL.

The likelihood for shear-related platelet activation is currently estimated based on instantaneous wall shear near the tears. In the tear regions, cells are generally exposed for short periods of time to high shear rates, often above the defined threshold of 10,000 s$^{-1}$, whereas shear rates in the other regions are usually at least one order of magnitude below the threshold. This has led to the assumption that in aortic dissection the peak shear stress on the tears will have a major impact on the activation process. A more comprehensive and more robust model which includes the effects of exposure time as well as shear loading history (Bluestein et al. 1997; Nobili et al. 2008) would be desirable to evaluate the likelihood for shear-related platelet activation. Also, experimental evidence shows that high shear rates could potentially lead to local platelet aggregation through the action of von Willebrand factor (vWF) (Bark et al. 2012; Ruggeri and Jackson 2013; Casa and Ku 2014). The high-shear thrombotic pathway is neglected in our current model, but it will be interesting to incorporate this pathway in our future work.

The model presented here aims to reproduce major changes in the flow domain by using simplified 2D models and neglects morphological changes that can take place in vivo. The simulated time frame of 20 s is not representative of the real time scale in patient-specific dissections, where long periods of weeks/months are required to observe FL thrombosis, depending on morphological features and treatment strategies adopted. This approach allows us to observe major qualitative changes caused by thrombus growth to the flow field, although neglecting actual growth rates and realistic time-scales of the process. Careful validations with patient-specific follow-ups will be needed to evaluate the impact of our assumptions, in particular neglecting the time-scales of biochemical reactions and speeding up the growth process by neglecting the effects of convection on the coagulation cascade. These will allow us to test the predictive power of the model and understand to what extent thrombus formation can be reliably represented.

Several assumptions have been made in the development of the phantom models. The use of 2D rigid geometries does not allow full representation of the complexity of flow in aortic dissection, which usually involves highly disturbed flow patterns, characterised by asymmetric helices, secondary motion, and regions of strong recirculation. Additionally, the presence of aortic branches and the compliant nature of aortic walls have an important role in modulating velocities and flow distribution between true and false lumen. The application of the model to patient geometries and further fluid-structure interaction (FSI) studies will allow us to study the impact of more realistic features on thrombosis in the false lumen.

## Conclusions

This paper presents the application of a hemodynamics-based model to the study of thrombosis in Type B aortic dissection under realistic flow conditions. The model makes use of key variables such as local shear rates and residence time to predict the formation of thrombus over time. The model was applied to different idealized aortic dissections in order to study the effect of geometric features on thrombosis, highlighting a potential association between size and location of tears and the formation of thrombus in the false lumen. A higher probability for complete thrombosis of the false lumen was predicted for dissections presenting only one proximal or distal tear, while in cases with multiple tears thrombus formation was confined to specific regions of the FL. Our study showed that smaller tears could increase the likelihood for thrombosis as they produce higher shear stress on the tears, promoting platelet activation, and favour the formation of extended recirculations zones. The effect of varying the distance between proximal and distal tears was also studied. Although a larger distance between tears was associated with higher local shear stress and with flow recirculation in the middle region of the FL, a shorter distance between tears resulted in more disorganized flow patterns in-between the tears, increasing the probability for thrombosis in this region. Our results show good qualitative agreement with previous in-vivo observations, highlighting the potential applicability of such a modeling approach to predict thrombosis in realistic geometries. Future work will involve the application of the presented model to patient-specific case studies, in order to validate the model and investigate the impact of the assumptions made.
